# Substrate Promiscuity of *Thermoplasma acidophilum* Malic Enzyme for CO_2_ Fixation Reaction

**DOI:** 10.1021/jacsau.4c00290

**Published:** 2024-05-14

**Authors:** Yuri Oku, Tomoko Matsuda

**Affiliations:** Department of Life Science and Technology, Tokyo Institute of Technology, 4259 Nagatsuta-cho, Midori-ku, Yokohama, 226-8501, JAPAN

**Keywords:** CO_2_ fixation, enzymatic carboxylation, malic enzyme, glucose dehydrogenase, CCU

## Abstract

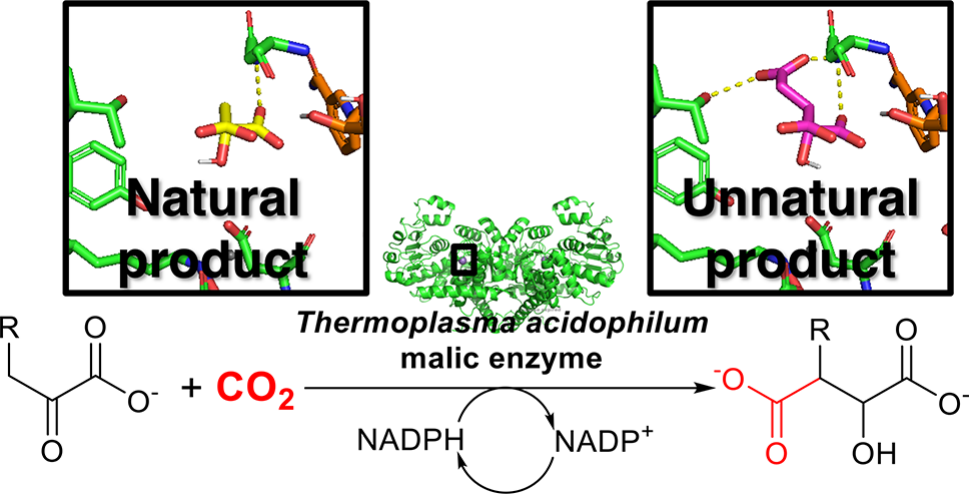

CO_2_ fixation technology has gained attention as a method to effectively utilize the abundant CO_2_ in the atmosphere by converting it into useful chemicals. However, since CO_2_ is a highly stable molecule, many of the currently developed methods for chemical CO_2_ fixation require harsh conditions and reactive reagents. The establishment of efficient and sustainable processes is eagerly awaited. In this study, we investigated a biocatalytic process and achieved a carboxylation reaction under mild conditions (37 °C, 0.1 MPa CO_2_) using a biocatalyst, *Thermoplasma acidophilum* NADP^+^-malic enzyme (*Ta*ME), and gaseous CO_2_ by coupling enzymatic coenzyme regeneration. We also demonstrated for the first time that the carboxylation reaction by ME proceeds not only with pyruvate, a natural substrate, but also with 2-ketoglutarate.

In recent years, due to the urgent need for decarbonization, Carbon Capture and Utilization (CCU) technology has garnered attention for its effective utilization of CO_2_, one of the primary contributors to global warming. Therefore, chemists have been developing reactions in which CO_2_ is fixed to organic molecules as carboxyl groups, as one of the CCU technologies.^1−5^ The attractiveness of utilizing CO_2_ as a reactant stems not only from its ability to promote CO_2_ utilization and produce value-added chemicals but also from its safety and ease of handling. For example, some carboxylic acids, such as acrylic acid and methacrylic acid, which are important compounds of pharmaceuticals and chemical synthesis, can be produced by carboxylation of olefins.^6^ However, given the unreactive nature of CO_2_, many of the currently developed methods for carboxylation reactions with CO_2_ as a reactant require high temperatures, pressures, and unstable reagents, posing challenges in terms of energy efficiency and sustainability. To address these challenges, the use of biocatalysts holds promise as they can catalyze reactions under mild conditions, produce minimal byproducts, and remain sustainable in the long term.^7−13^

Hence, there have been reports of some biocatalytic reactions involving CO_2_ fixation.^14^ For instance, 2,3-dihydroxybenzoic acid decarboxylase has been noted for its role in catalyzing the carboxylation reaction of catechol with gaseous CO_2_, coupling with simultaneous amine-mediated conversion of CO_2_ to bicarbonate.^15^ Enzymes within the pyridine nucleotide-linked hydroxy acid oxidative decarboxylases superfamily, including “β-decarboxylating dehydrogenases” and “malic enzymes (MEs)” families, have also been identified as catalysts for fixing CO_2_ to α-ketoacid.^16−18^ For example, *T. acidophilum* isocitrate dehydrogenase (*Ta*IDH) was employed in the carboxylation of 2-ketoglutarate to isocitrate under 10 MPa CO_2_.^18^ Additionally, malic enzyme (ME), known as a catalyst for the oxidative decarboxylation reaction of l-malate, producing pyruvate along with the reduction of NAD(P)^+^, also catalyzes the thermodynamically very unfavorable reverse reaction, the reductive carboxylation. This thermodynamic constraint was overcome by combining electrochemical, photochemical, and enzymatic reaction systems. These systems regenerate the reduced form of cofactors (NAD(P)H), which shifts the equilibrium toward carboxylation.^18−21^ However, only a handful of studies have reported ME-catalyzed carboxylation using only gaseous CO_2_ as CO_2_ source;^22^ most biocatalytic carboxylations use bicarbonate such as NaHCO_3_ and KHCO_3_. Furthermore, researchers have explored few substrates other than malate **1b** and pyruvate **1a**, which are natural substrates for decarboxylation and carboxylation reactions, respectively, to determine if they serve as substrates for ME. In this study, our focus was on the NADP^+^-malic enzyme from *Thermoplasma acidophilum* (*Ta*ME) (Scheme 1). We chose this enzyme hoping for its robustness and ease of handling, as other enzymes from *T. acidophilum*, such as isocitrate dehydrogenase (*Ta*IDH) and glucose dehydrogenase (*Ta*GDH), were reported to have high thermal and CO_2_-pressure stabilities.^18^ Additionally, *Ta*GDH was employed to address the thermodynamic constraint for carboxylation. The objective of our study was to develop a *Ta*ME-catalyzed carboxylation reaction using only gaseous CO_2_ as a CO_2_ source and to investigate the substrate specificity of *Ta*ME for both carboxylation and decarboxylation.

**Scheme 1:**
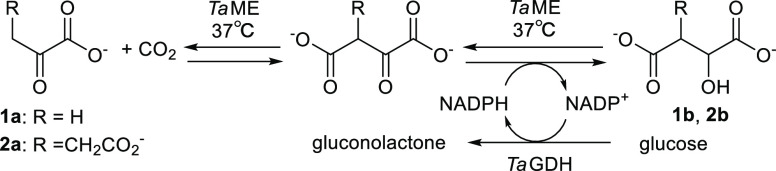
*Ta*ME-Catalyzed Carboxylation with Coupled *Ta*GDH-Catalyzed NADPH Regeneration

Initially, the carboxylation of pyruvate **1a** was conducted using *Ta*ME and NADPH under 0.1 MPa of CO_2_. While previous studies often relied on carbonates such as NaHCO_3_ or KHCO_3_ as CO_2_ sources (Table S2), our study exclusively used gaseous CO_2_. The resulting yield of the reaction by *Ta*ME and a molar equivalent of NADPH to the substrate was 3.8%, as shown in Figure 1a. To address this low yield, we explored a cofactor regeneration system, which involved the addition of *Ta*GDH and d-glucose. This addition led to a substantial improvement, with the yield increasing to 67%, an 18-fold increase (Figure 1a). This result indicated that the *Ta*GDH reaction enhanced the thermodynamic feasibility of the carboxylation reaction, aside from cofactor regeneration, as expected from the case using *Ta*IDH and *Ta*GDH.^18^

**Figure 1:**
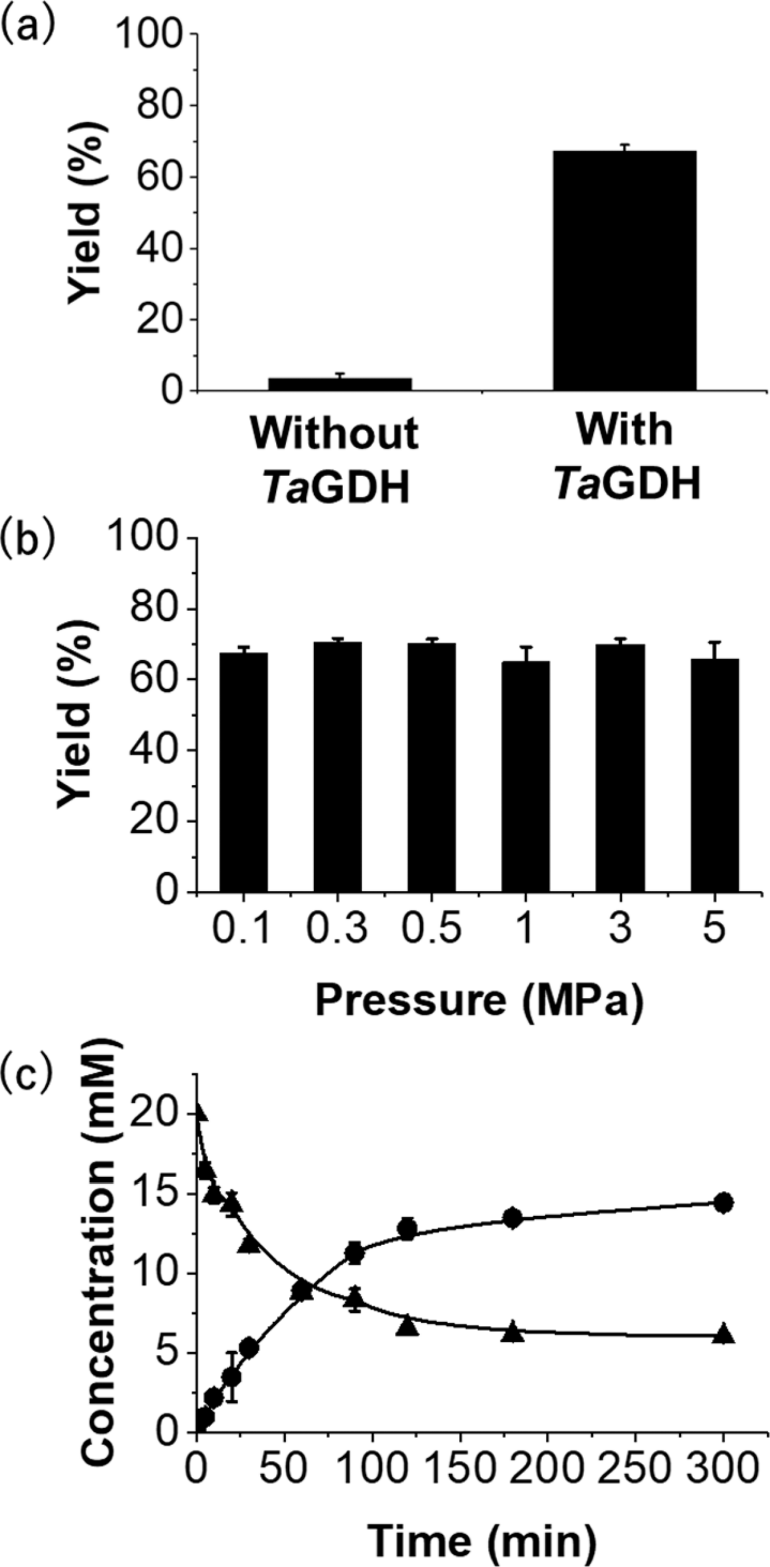
Investigation of conditions for *Ta*ME-catalyzed carboxylation of **1a**: (a) effect of cofactor regeneration, (b) effect of CO_2_ pressure, and (c) time course (circle: product conc., triangle: substrate conc.) The yield was determined by the method in Supporting Information section 1.4. For the reaction without *Ta*GDH, NADPH was added instead of glucose and *Ta*GDH.

Then, we investigated the reaction conditions for the reductive carboxylation of **1a** by *Ta*ME, gaseous CO_2_, and *Ta*GDH. The effects of pH, divalent metal ion, substrate concentration, and CO_2_ pressure on the carboxylation reaction were investigated. As shown in Figure S2a, the optimal yield was achieved at an initial pH of 7.5–8.5, with a decline observed at pH 7.0. Notably, this pH range differs from the reported optimal pH for the reductive carboxylation reaction by ME from *Pseudomonas diminuta* IFO 13182 (pH 6.0)^20^ and *Thermococcus kodakarensis* (pH 6.5)^23^ as well as the reported *Ta*GDH activity for NADPH production (pH 6.5).^24^ The differences in optimal pH values may be attributed to the likelihood that the actual pH during the reaction in our experiment was lower than the initial pH due to the formation of H_2_CO_3_ and subsequent pH reduction. Considering this factor, the optimum pH for carboxylation with *Ta*ME was set at 7.5.

Next, the effect of divalent metal ions was examined using chloride salts of Mg^2+^, Mn^2+^, Co^2+^, Ca^2+^, and Ni^2+^, as these ions (mainly Mn^2+^ and Mg^2+^) are crucial for ME catalysis. They help bind substrates to the active site with stability and prevent the dissociation of ME subunits, an oligomeric enzyme.^25−27^ Consequently, we found that the highest yields for carboxylation with *Ta*ME were achieved with Mg^2+^, Mn^2+^, and Co^2+^ (Figure S2b). Therefore, we selected Mg^2+^, which has the lowest environmental impact among these metals, as the optimal divalent metal ion for carboxylation.

Then, the effect of substrate concentration (10, 20, 30, and 40 mM) was examined. The product concentration increases with increasing substrate concentration but not the yield, as shown in Figure S2c. Therefore, considering both the final product concentration and yield, we set 20 mM as the optimal concentration. These conditions (pH 7.5, Mg^2+^, and 20 mM substrate) were used thereafter in *Ta*ME carboxylation reactions.

Furthermore, the effect of CO_2_ pressure on the reductive carboxylation of **1a** by *Ta*ME was investigated, and no significant difference in yield was observed between 0.1 to 5 MPa gaseous CO_2_, as shown in Figure 1b. The solubility of CO_2_ at 303 K, which is close to the experimental temperature (310 K), was reported to be 28.6 mM (0.1 MPa), 280 mM (1 MPa), and 1080 mM (5 MPa).^28^ On the other hand, it was reported that *K*_*m*(CO2)_ was 4.2 mM for the wheat germ preparation and 1.1 mM for the C4 plant enzyme.^29,30^ Therefore, the insignificant difference in yield between 0.1 and 5 MPa was attributed to the fact that even at 0.1 MPa CO_2_, the concentration of CO_2_ was assumed to be sufficiently higher than the *K*_*m*(CO2)_ values of *Ta*ME. Lastly, the time course of the reductive carboxylation of **1a** by *Ta*ME under 0.1 MPa CO_2_ was examined. Figure 1c shows that the yield increased steadily over time and reached a plateau after 5 h, with a remarkable yield of 72%, which was the highest of any carboxylation reaction with ME developed in the past (Table S2).

Next, we investigated substrate specificity of *Ta*ME for decarboxylation and carboxylation. First, the decarboxylation activity was determined using malate **1b**, isocitrate **2b**, citrate **3b**, 3-isopropylmalate **4b**, tartrate **5b**, tartornate **6b**, and lactate **7b** each at a concentration of 1 mM by monitoring the increase in NADPH concentration, setting the activity toward **1b**, the natural substrate of *Ta*ME, as 100% (Table 1). Surprisingly, *Ta*ME exhibited higher relative activity (190%) toward **2b** compared to **1b**. To explain these findings, Michaelis–Menten kinetic parameters for the decarboxylation of **1b** and **2b** were determined (Table 2). The results revealed that *k*_cat_ for **2b** (1.3 s^–1^) was about twice that of **1b** (0.69 s^–1^). Additionally, the *K*_*m*_ for **2b** (23.7 μM) was significantly lower than the *K*_*m*_ for **1a** (879 μM). Since the substrate concentration used to determine the activity was 1 mM, both higher *k*_cat_ and lower *K*_*m*_ of **2b** likely contributed to its higher relative activity of **2b** than that of **1b**. It was surprising that the *k*_cat_/*K*_*m*(substrate)_ values differed by 2 orders of magnitude, with superiority in the unnatural substrate **2b**.

**Table 1:**
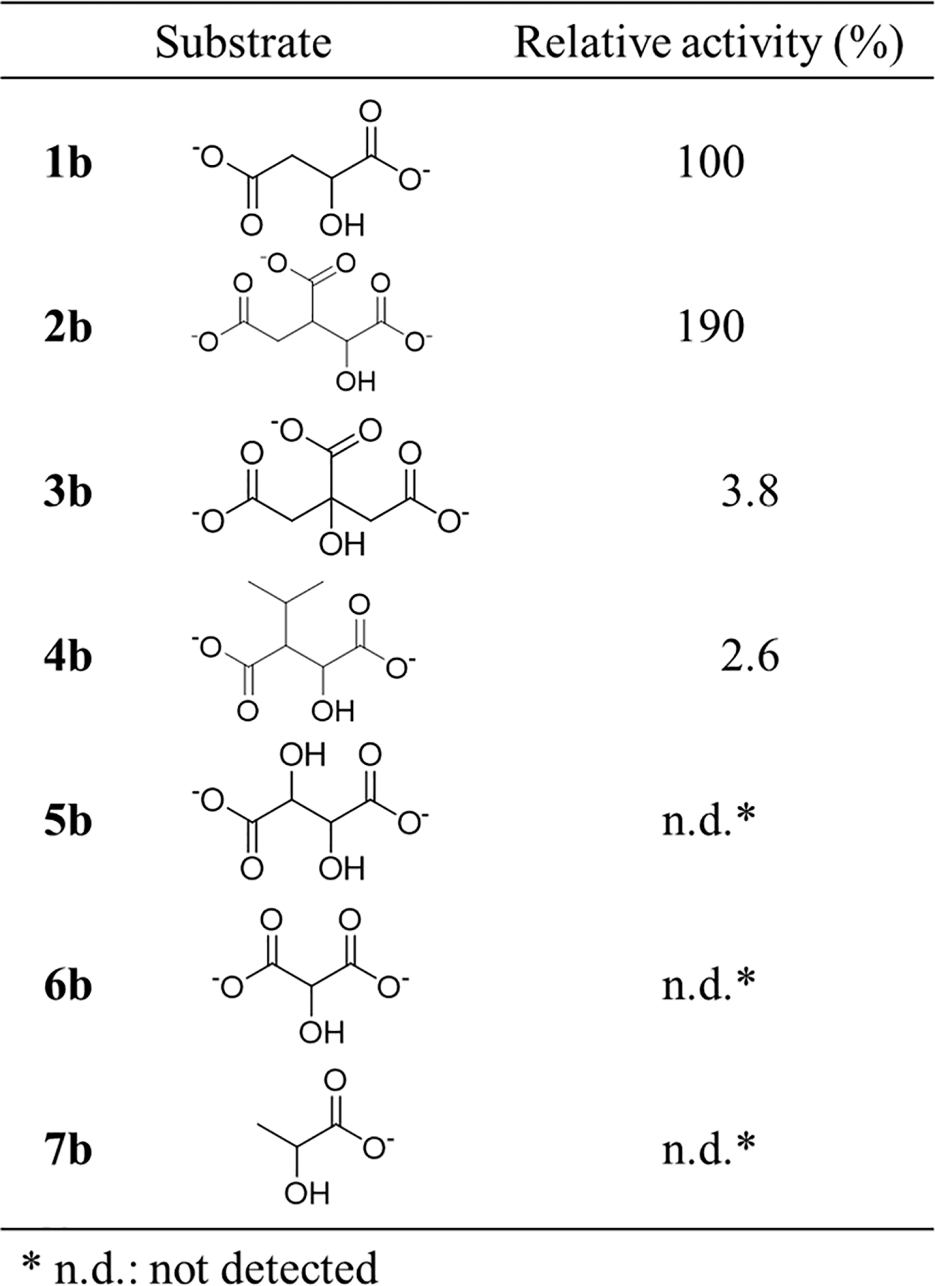
Substrate Specificity for *Ta*ME-Catalyzed Decarboxylation^a^

a The activity was determined by the method in Supporting Information section 1.3. The activity toward malate **1b** was set to be 100%.

**Table 2:** Michaelis–Menten Kinetic Parameters of *Ta*ME-Catalyzed Decarboxylation of **1b** and **2b**^a^

Substrate		*k*_cat_ (s^–1^)	*K*_*m*(substrate)_ (μM)	*k*_cat_/*K*_*m*(substrate)_ (M^–1^ s^–1^)
**1b**	Malate	0.69 ± 0.06	879 ± 149	7.8 × 10^2^
**2b**	Isocitrate	1.3 ± 0.3	23.7 ± 5.2	5.5 × 10^4^

a Kinetics assays were done under standard assays conditions shown in the Supporting Information section 1.3.1 using 0.05–1.0 mM of **1b** or 0.03–0.60 mM of **2b**. The *Ta*ME activity toward **2b** reached a plateau after 0.6 mM up to 2.0 mM.

To further investigate the differences in the relative activity and Michaelis–Menten kinetic parameters of *Ta*ME between **1b** and **2b**, docking simulations were performed. The X-ray structure of ME from *Bdellovibrio bacteriovorous* (Bd1833/MaeB), containing NADP^+^ and Mg^2+^ (6ZN7.pdb),^31^ was used first to dock **1b** and **2b** using Autodock Vina due to the high amino-acid sequence homology with *Ta*ME (Figure S3). Then, the resulting structures were aligned with the structure of *Ta*ME predicted by ColabFold using PyMOL (Figure S4a, b), and the interactions between the substrates (**1b** and **2b**) and the *Ta*ME were investigated (Figure 2). Consequently, **2b** formed three hydrogen bonds (defined within 3.5 Å) with amino acid residues Asn304 and Thr46* (the asterisk indicates residues from another subunit of the dimer) of *Ta*ME (Figure 2b), whereas **1b** formed only one hydrogen bond with Asn304 (Figure 2a). Therefore, it is assumed that the difference in the number of hydrogen bonds contributed to the difference in *k*_cat_/*K*_*m*_. Some previous studies also reported similar hydrogen bonds between IDH and isocitrate; the hydrogen bond formed between Ser113, Thr105, and Asn115 of IDH from *Escherichia coli* and the γ-carboxylate of bound isocitrate was essential to determine the substrate specificity.^32−34^ Therefore, Asn304 and Thr46* of *Ta*ME may also be important residues in determining the substrate specificity. However, there are critical differences between the 3D arrangements of ME and IDH, which are enantiomeric. The active site of ME is the mirror image of that of IDH, and this is consistent with the opposite stereochemistry at the C_α_ alcohol of l-malate, the natural substrate of ME, and d-isocitrate, the natural substrate of IDH.^35^

**Figure 2:**
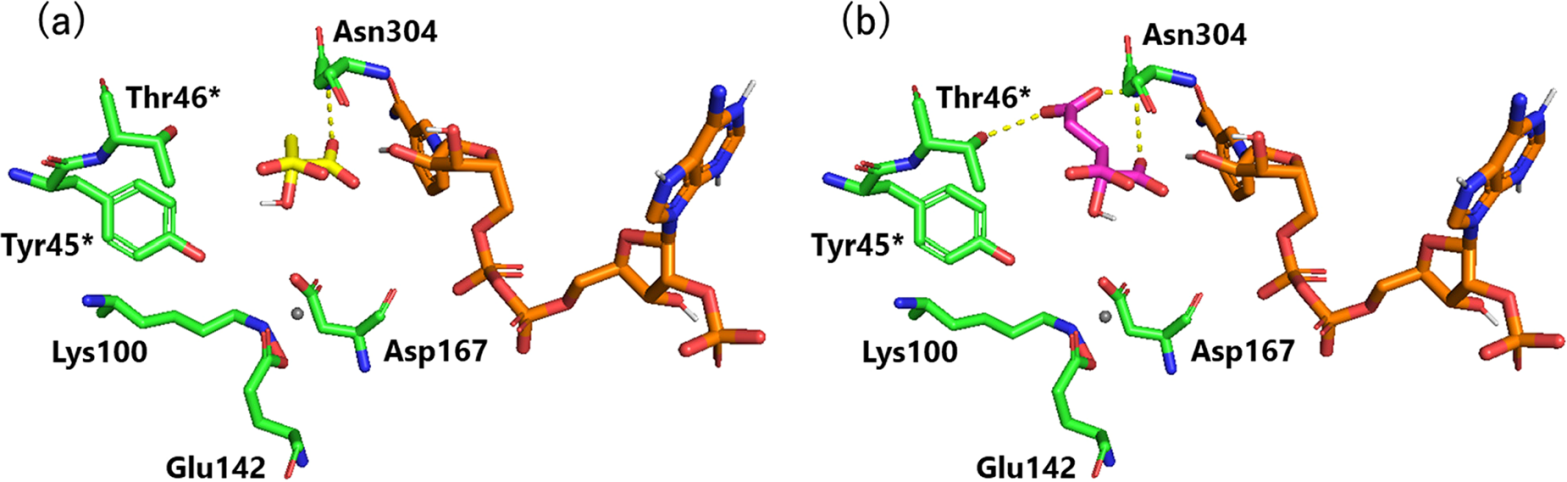
Active site structure of *Ta*ME predicted by ColabFold with (a) malate **1b** and (b) isocitrate **2b**; *Ta*ME: green, **1b**: yellow, **2b**: magenta, Mg^2+^: gray, NADP^+^: orange, hydrogen bond: dashed yellow line.

Encouraged by the higher activity of *Ta*ME toward unnatural substrate **2b**, finally, reductive carboxylation of 2-ketoglutarate **2a** was investigated to synthesize **2b** with high added value.^36^ This involved the reaction of **2a** in the presence of *Ta*ME, 0.1 MPa CO_2_, Mg^2+^, NADP^+^, *Ta*GDH, and glucose in HEPES buffer for 3 h, resulting in the successful synthesis of isocitrate **2b** with a yield of 27%. This low yield could be attributed to the accelerated rate of the decarboxylation reaction of **2b**, as can be seen in the notably larger *k*_cat_/*K*_*m*_ of **2b** in comparison to **1b**. Future study to examine reaction conditions and time is necessary to improve the yield. The absolute configuration of the product **2b**, which will be determined in the future study, may be l-(*S*), the opposite enantiomer of what is produced by the carboxylation using *Ta*IDH.^18^ This is because *Ta*ME, belonging to the “malic enzymes” family, accepts (*S*)-hydroxy acids as substrates, while IDH, belonging to the “β-decarboxylating dehydrogenases” family, accepts (*R*)-hydroxy acids as substrates.^35^

It is noteworthy that *Ta*ME was found to exhibit the substrate promiscuity for both decarboxylation and carboxylation in this study, as it belongs to the “malic enzymes” family.^35,37^ While enzymes in the “β-decarboxylating dehydrogenases” family (IDH, isopropyl malate dehydrogenase, homoisocitrate dehydrogenase, d-malate dehydrogenase, and tartrate dehydrogenase) generally have narrow substrate specificities,^33,38^ some enzymes in this family have been reported to catalyze secondary reactions.^17,34^ However, very few investigations have been performed on the substrate specificity of decarboxylation and carboxylation for the “malic enzymes” family. Thus, this study, which demonstrates substrate promiscuity not only for decarboxylation but also for carboxylation, opens up the possibilities that other enzymes in the “malic enzymes” family, complementary to those in the “β-decarboxylating dehydrogenases” family, would also possess catalytic promiscuity and could be utilized for wide applications in carboxylation.

In conclusion, we found that *Ta*ME was a significant biocatalyst for carboxylation under mild conditions, contributing to Carbon Capture and Utilization (CCU). We demonstrated that *Ta*ME could catalyze the carboxylation of not only pyruvate **1a**, a natural substrate, but also unnatural substrate 2-ketoglutarate **2b**. Since the hydrophilic amino acid residues such as Asn304 and Thr46* of *Ta*ME were suggested to be important in determining substrate specificity, it is expected that introducing site-directed mutations in these residues will further expand the substrate specificity in the future studies. Additionally, *Ta*ME may potentially catalyze the selective synthesis of (*S*)-hydroxy acids, which could be complementary to our previously developed carboxylation reaction with *Ta*IDH.^18^

## Supporting Information Available

Additional experimental details, materials and methods, and additional results, including the SDS-PAGE of *Ta*ME (Figure S1), purification fold table of *Ta*ME (Table S1), effect of initial pH, divalent metal ion, and substrate concentration on *Ta*ME-catalyzed carboxylation of **1a** (Figure S2), amino acid sequence alignment of *Ta*ME and Bd1833/MaeB (Figure S3), overlapping of *Ta*ME ColabFold structure with docking simulation results of Bd1833/MaeB crystal structure with malate **1b** and isocitrate **2b** (Figure S4), and an example of reductive carboxylation of **1a** by ME (Table S2) (PDF)
