# Supplementary material for: Substrate Promiscuity of Thermoplasma acidophilum Malic Enzyme for CO2 Fixation Reaction
Source: JACS Au. 2024 May 14;4(5):1758–62. doi: 10.1021/jacsau.4c00290 (PMC11134350; doi:10.1021/jacsau.4c00290)
Supplement: Supplementary file 1 — au4c00290_si_001.pdf [file au4c00290_si_001.pdf]

Supporting information

Substrate Promiscuity of *Thermoplasma acidophilum* Malic Enzyme for CO_2 Fixation Reaction

Yuri Oku, Tomoko Matsuda*

Department of Life Science and Technology, Tokyo Institute of Technology, 4259 Nagatsuta-cho, Midori-ku, Yokohama, 226-8501, JAPAN.

tmatsuda@bio.titech.ac.jp

1.	Material & method	2
1.1.	Equipment and reagents	2
1.2.	Cloning and overexpression of *Ta*ME and preparation of *Ta*GDH	2
1.3.	Activity assay	3
1.3.1.	*Ta*ME decarboxylation activity assay	3
1.3.2.	*Ta*GDH activity assay	3
1.4.	Carboxylation	3
1.4.1.	Carboxylation reaction procedure	3
1.4.2.	Analytical method to determine the yield of carboxylation	4
1.5.	Simulation	4
2.	Supplementary material	
Fig. S1	SDS PAGE of *Ta*ME	5
Table S1	Purification of *Ta*ME by heat treatment	5
Fig. S2	Effect of (a) initial pH (square: MES-KOH, circle: HEPES-KOH, triangle: Glycine-KOH), (b) divalent metal ion, and (c) substrate concentration (circle: yield, square: product concentration) on *Ta*ME catalyzed carboxylation of **1a**	5
Fig. S3	Amino acid sequence alignment of *Ta*ME and Bd1833/MaeB (6ZN7.pbd)	6
Fig. S4	Overlapping of *Ta*ME ColabFold structure with docking simulation results of Bd1833/MaeB crystal structure with (a) malate **1b** and (b) isocitrate **2b**	6
Table S2	Examples of reductive carboxylation of **1a** by $NAD(P)^+$-malic enzymes	7
References		8

1. Material & method

1.1. Equipment and reagents

The polymerase chain reaction (PCR) was carried out using GeneAmp PCR System 9700 from Applied Biosystems (USA). Cell disruption was done using Ultrasonic Generator (Insonator 201 M) from Kubota (Tokyo, Japan). The UV-spectroscopic analysis was done using UV-1900-UV–vis spectrophotometer from Shimadzu (Kyoto, Japan). The HPLC analysis was done using HPLC (Nexera Organic Acid Analysis System from Shimadzu (Kyoto, Japan), equipped with a Shim-pack SCR-102H column. All high-pressure experiments were conducted using 10 mL of SUS316 pressure-resistant vessel from Taiatsu Techno (Tokyo, Japan). Reagents were purchased from Nacalai Tesque, Inc. (Japan) or Wako Pure Chemical Industries, Ltd. (Japan) and used without purification unless indicated otherwise. Genomic DNA of *Thermoplasma acidophilum* ATCC 25905D-5 for the cloning of *Ta*ME was purchased from the National Institute of Technology and Evaluation.

1.2. Cloning and overexpression of *Ta*ME and preparation of *Ta*GDH

*Ta*ME was cloned and overexpressed using a similar method as previously reported.[1] *Ta*ME gene (Ta0456m, KEGG) was amplified by PCR using commercially available purified genomic DNA of *T. acidophilum* ATCC, 25905D-5 using the following primers: FW (5′ - TTGCTATACGGTGTTTGTCGT - 3′) and RV (5′ - CAGCAGTACATGGGAATCGT - 3′). Gene encoding *Ta*ME was inserted into pET21b(+) and transformed to Rosetta™(DE3)pLysS. Antibiotics used for cultivation were both 125 μg/mL carbenicillin and 20 μg/mL chloramphenicol. Rosetta™(DE3)pLysS-pET21b(+)-*Ta*ME was pre-cultured in 5 mL Luria-Bertani (LB) medium at 37 °C and 250 rpm for 18 – 20 h. 2.5 mL of pre-cultured *E. coli* were transferred to 250 mL LB medium and cultured until OD_{600} reached 0.5−0.7. Then, 2.5 mL of 100 mM isopropyl β-D-1-thiogalactopyranoside (IPTG) was added, and it was cultured for 18-20 h at 20 °C and 250 rpm, centrifuged at 10000 G for 5 min, washed with 50 mL of 0.85 % (m/v) NaCl solution, centrifuged at 10000 G for 5 min, and homogenized with 10 mL of 100 mM KH_2PO_4-K_2HPO_4 buffer (pH 7) with 1 mM 1,4-dithiothreitol (DTT), and 1 mM phenylmethylsulfonyl fluoride (PMSF). Then, the solution underwent an ultrasonic lysis treatment (100 W, 30 min, 0 °C). The lysate was centrifuged at 12000 G for 30 min at 4 °C. The supernatant was heat-treated at 60 °C for 10 min and centrifuged at 12000 G for 30 min. The protein was concentrated by ultrafiltration using Amicon Ultra-15 10-K MWCO (Merck). The protein was partially purified by 2.0 fold with 53.4% yield, resulting in the activity of 0.38 μmol/mg/min (Table S1), as shown by a band in SDS-PAGE (Fig. S1). Protein concentration was measured by the Bradford method. 5-20 times diluted *Ta*ME-enzyme solution was used for further studies.

*Ta*GDH (Ta0897, KEGG) was prepared according to a previously reported procedure using *E. coli* Rosetta™(DE3)pLysS-pET21b(+)-*Ta*GDH.[1]

1.3. Activity assay

Activities of *Ta*ME and *Ta*GDH were determined using a similar method as previously reported for *Ta*IDH and *Ta*GDH.[1]

1.3.1. *Ta*ME decarboxylation activity assay

940 μL of 100 mM 4-(2-hydroxyethyl)-1-piperazineethanesulfonic acid (HEPES)-KOH buffer containing 50 mM KCl (pH 6.5), 10 μL of 100 mM L-malate **1b**, and 20 μL of 200 mM $MnCl_2$ were mixed and incubated at 37 °C for 15 min. 10 μL of *Ta*ME solution and 20 μL of a 10 mM $NADP^+$ was added, and the change in absorbance at 340 nm was measured. One unit of enzyme activity was defined as the micromoles of NADPH released by the decarboxylation and oxidation of **1b** per minute under the above-mentioned conditions. The above assays were repeated using various substrates (DL-isocitrate **2b**, citrate **3b**, DL-3-isopropylmalate **4b**, tartrate **5b**, tartornate **6b**, and L-lactate **7b**). For the determination of Michaelis–Menten kinetic parameters of *Ta*ME catalyzed decarboxylation of **1b** and **2b**, the above assays were repeated using 0.05-1.0 mM of **1b** or 0.03-0.60 mM of **2b**.

1.3.2. *Ta*GDH activity assay

960 μL of 100 mM HEPES-KOH buffer containing 50 mM KCl (pH 6.5) and 10 μL of 100 mM D-glucose were mixed and incubated at 37 °C for 15 min. 10 μL of *Ta*GDH solution and 20 μL of 10 mM $NADP^+$ was added, and the change in absorbance at 340 nm was measured. One unit of enzyme activity was defined as the micromoles of NADPH released by the oxidation of D-glucose per minute under the above-mentioned conditions.

1.4. Carboxylation

1.4.1. Carboxylation reaction procedure

200 μL of 5 M D-glucose (final concentration of 1 M), 40 μL of 1 M $MgCl_2$ solution (final concentration of 40 mM), 50 μL of 10 mM $NADP^+$ (final concentration of 0.5 mM), 50 μL of 200 mM sodium pyruvate (final concentration of 20 mM), 1 U of *Ta*GDH solution, 0.5 U of *Ta*ME solution, and 500 mM HEPES buffer containing 100 mM KCl (pH 7.5) to make the total volume of 1 mL were mixed in pressure-resistant vessel (10 mL). Then, CO_2 was introduced until the pressure was 0.1 MPa. The solution was stirred by a magnetic stirrer for 3 h at 37 ◦C. The reaction was quenched by depressurization.

The above reactions were repeated at various pH (5.5, 6.5, 7, 7.5, 8.5, or 9.5), at various substrate concentrations (10, 20, 30, or 40 mM), using different metal salts ($MgCl_2$, $MnCl_2$, $CoCl_2$, $NiCl_2$, or $CaCl_2$), under various CO_2 pressure (0.1, 0.3, 0.5, 1, 3, or 5 MPa), or various reaction time (5, 10, 20, 30, 60, 90, 120, 180, or 300 min). 20 mM NADPH was added instead of glucose and *Ta*GDH for the reaction without *Ta*GDH. The effects of pH, metal ion, and substrate concentration on the carboxylation of **1a** were investigated at pH 7.0 using $MnCl_2$ at 1 MPa CO_2. The effects of CO_2 pressure and reaction time on the carboxylation of **1a** were investigated at pH 7.5 using $MgCl_2$ at 0.1

MPa CO_2. The carboxylation of **2a** was conducted at pH 7.5 using $MgCl_2$ at 0.1 MPa CO_2. 500 mM MES buffer containing 100 mM KCl was used for pH investigation at pH 5.5 and pH 6.5. 500 mM glycine buffer containing 100 mM KCl was used for pH investigation at pH 8.5 and pH 9.5.

1.4.2. Analytical method to determine the yield of carboxylation

The carboxylation yield was determined by HPLC analysis. A calibration curve was prepared as follows. At first, solution 1 and 2 were prepared.

Solution 1 (pyruvate **1a** and malate **1b)**: 280 mM HEPES-KOH buffer (pH 7.5) containing 56 mM KCl, 0.5 mM $NADP^+$, 40 mM $MgCl_2$, 10 mM KH_2PO_4-K_2HPO_4 buffer (pH 7), 0.1 mM DTT, 0.1 mM PMSF, 20 mM sodium pyruvate **1a**, and 20 mM L-malic acid **1b**

Solution 2 (2-ketoglutarate **2a** and isocitrate **2b)**: 280 mM HEPES-KOH buffer (pH 7.5) containing 56 mM KCl, 0.5 mM $NADP^+$, 40 mM MgCl2, 10 mM KH_2PO_4-K_2HPO_4 buffer (pH 7), 0.1 mM DTT, 0.1 mM PMSF, 25 mM disodium 2-ketoglutarate **2a**, and 25 mM trisodium DL-isocitrate **2b**

Then, 600 μL of solution 1 was mixed with 150 μL of 100% (w/v) trichloroacetic acid (TCA). The mixture was incubated at 4 °C for 15 min and centrifuged at 12000 G, 0 °C for 5 min. The supernatant was filtered (0.2 μm aperture) and diluted with distilled water to adjust the concentration of pyruvate **1a** and malate **1b** to be 0.4, 0.8, 1.2. 1.6, and 2.0 mM (final concentration). The above procedure was also repeated for solution 2 to adjust the concentration of 2-ketoglutarate **2a** and isocitrate **2b** to be 0.5, 1.0, 1.5, 2.0, and 2.5 mM (final concentration). These standard solutions were used to prepare the calibration curve for the HPLC analysis.

The yield of the carboxylation reaction was determined as follows. 600 μL of the carboxylation reaction mixture was mixed with 150 μL of 100% (w/v) TCA to precipitate protein. The mixture was incubated at 4 °C for 15 min and centrifuged at 12000 G, 0 °C for 5 min. The supernatant was filtered (0.2 μm aperture), 8-30 times diluted with distilled water, and used for the HPLC analysis. The yield was calculated according to the following equation.

$$Yield = \frac{Concentration\ of\ malate\ \mathbf{1b}\ (or\ isocitrate\ \mathbf{2b})\ after\ reaction}{Initial\ concentration\ of\ pyruvate\ \mathbf{1a}\ (or\ 2-ketoglutarate\ \mathbf{2a})} \times 100$$

1.5. Simulation

Docking simulations were done for 2 ligands, malate **1b** and isocitrate **2b**, with the malic enzyme from *Bdellovibrio bacteriovorous* (Bd1833/MaeB)-$NADP^+$-Mg^{2+} complex crystal structure (6zn7.pdb)[2] as the receptor. The sdf files for ligands were obtained from PubChem and converted to pdb format. Receptors and ligands were prepared using AutoDockTools 1.5.6, and the docking was done by using AutoDock Vina. The size of a cubic gridbox centered on the receptor chain A catalytic magnesium was set to 1000 Å. The pdb file of the *Ta*ME-homodimer structure was predicted by ColabFold v1.5.5. The ligand-docked structures resulting from the simulation using (Bd1833/MaeB)-$NADP^+$-Mg^{2+} complex crystal structure were aligned with the structure of *Ta*ME by PyMOL.

2. Supplementary material

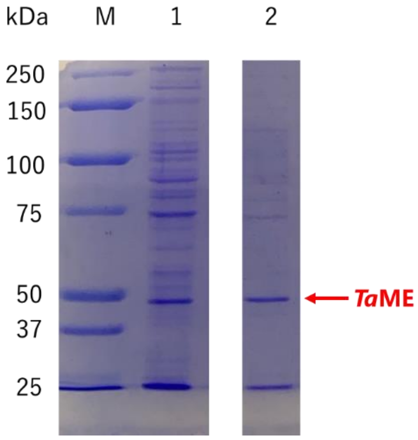

Fig. S1 SDS PAGE of *Ta*ME. Lane M, molecular weight standards (Precision Plus Protein™ All Blue Standards); lane 1, cell-free extract of *Ta*ME; lane 2, heat-treated *Ta*ME (5 µL of 10 times diluted cell free extract for lane 1 and 5 µL of 8 times diluted heat-treated cell free extract for lane 2 were applied.)

Table S1 Purification of *Ta*ME by heat treatment

	Total protein (mg)	Total unit (U)	Specific activity (U/mg)	Yield (%)	Purification fold
Cell-free extract	59.6	11.4	0.19	100	1.0
Heat-treated	15.9	6.09	0.38	53.4	2.0

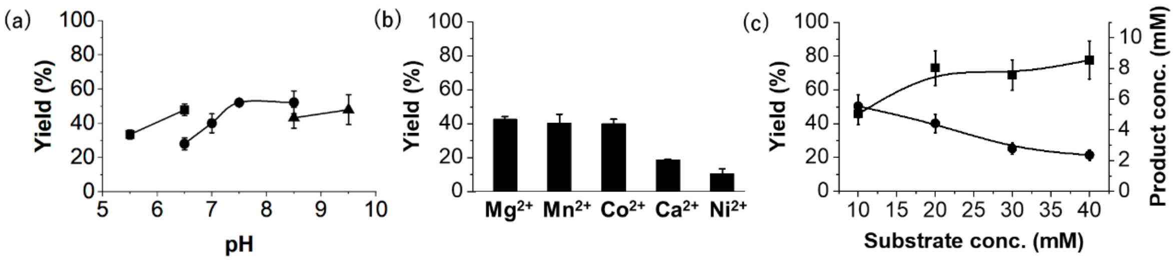

Fig. S2 Effect of (a) initial pH (square: MES-KOH, circle: HEPES-KOH, triangle: Glycine-KOH), (b) divalent metal ion, and (c) substrate concentration (circle: yield, square: product concentration) on *Ta*ME catalyzed carboxylation of **1a**. The yield was determined by the method in supporting information section 1.4.

```
TaME  IEPKTGTTNFDQEALLYHQQGKPGKIEVISSKPCATEKDLSLAYSPGVAAPCKAIAKDPA  79
      +E +  T  ++++AL Y +  K GKI+ IS  P  +  D S+ Y+PGVAA  + IA DP
6ZN7  MEDQERTERYNEDALRYSEYFK-GKIQTISKVPVRSLDDFSIWYTPGVAAVSRKIASDPD  61

TaME  KVYDYTAKGNLVAVISNGTAVLGLGNIGPAAGKPVMEGKGILFKQFAGIDVFDIEVAATD  139
        ++ T + N +A++++GT VLGLGNIGP A  PVMEGK ++F    G++   I +
6ZN7  LSFELTGRWNSIAILTDGTRVLGLGNIGPEAAMPVMEGKALIFNYLGGVNAIPIPIRVQS  121

TaME  VDVFCNAVRVLEPTFGGINLEDIKAPECFEIEERLKKEMNIPVFHDDQHGTAIVSGAALL  199
       + F    + LEP+FGGINLEDI++P+CF + E L+ EMNIPV+HDDQ GTA ++ A ++
6ZN7  KEEFVKVAKALEPSFGGINLEDIESPKCFFLLETLQNEMNIPVWHDDQLGTASITLAGVI  181

TaME  NACSITNRKMETVRIVVNGAGASANSCAKIFIALGARRENIIMCDSQGVIYKGRT---AG  256
      N+  I  +K++ V++V NGAGA+  + A +F A G + +N+I+ DS+G+++  R   A
6ZN7  NSLRIVGKKIDQVKVVFNGAGAANIAAAYLFKAAGFKMKNMILVDSKGILHPEREDIDAL  241

TaME  M--NKYKEYFASETEART----LTEALRGADVFVGLSVAG--ALTPEMLKDMAKDPIIFA  308
      M  N +K   A ET        L+ A+ GAD+ +  + +G   +   ++K M  D I+F
6ZN7  MINNPWKYQLAIETNGERVKGDLSNAIEGADLLISAAKSGPDTIDSRIVKKMNNDAIVFV  301

TaME  MANPEPEITPDKAR--AARPDAIIATGRSDYPNQVNNVLGFPSIFRGALDTRSTQINEEM  366
      +ANP PE+ P +A+   AR   I+ATGR D+PNQ+NN L FP +FRG LD R+  +N ++
6ZN7  LANPIPEMWPQEAKENGAR---IVATGRGDFPNQINNSLVFPGVFRGVLDARAKGVNFDV  358

TaME  KLAAVHALAKLAREDVPDKVSAT  389
       +AA + +A    +   +K+  T
6ZN7  MVAASYEIANFVEDPTEEKIVPT  381
```

Fig. S3 Amino acid sequence alignment of *Ta*ME and Bd1833/MaeB (6ZN7.pbd)[2]

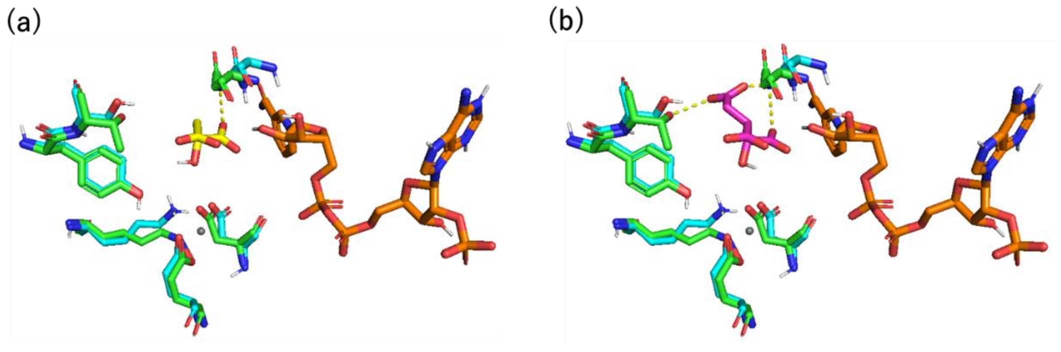

Fig. S4 Overlapping of *Ta*ME ColabFold structure with docking simulation results of Bd1833/MaeB crystal structure with (a) malate **1b** and (b) isocitrate **2b** (Bd1833/MaeB: cyan, *Ta*ME: green, **1b**: yellow, **2b**: magenta, Mg^{2+}: gray, $NADP^{+}$: orange, hydrogen bond: dashed yellow line)

Table S2 Examples of reductive carboxylation of **1a** by NAD(P)$^+$-malic enzymes

Enzyme source	pH	Divalent metal ion	CO_2 source	Yield (%)	Product conc. (mM)	Reference
Chicken liver	7.9	Mn^{2+}	$NaHCO_3$, CO_2	-	-	3
Chicken liver	6.0	Mn^{2+}	Saturated CO_2	-	-	4
Chicken liver	7.0	-[a]	Saturated CO_2 $NaHCO_3$	48	0.48	5
Chicken liver	7.4	Mg^{2+}	$NaHCO_3$	18.5	0.15	6
Pseudomonas diminuta IFO 13182	7.4	Mg^{2+}	$KHCO_3$	38	40	7
Chicken liver	8.0	-[a]	$NaHCO_3$	20	2.0	8
Brevundimonas diminuta IFO13182	7.4	-[a]	$KHCO_3$	2.4	11.9	9
Thermococcus kodakarensis	7.0	Mn^{2+}	$NaHCO_3$, CO_2	62	0.62	10
Thermococcus kodakarensis	7.0	Mn^{2+}	$NaHCO_3$, CO_2	17~20	5~6	11
Escherichia coli	7.5	Mg^{2+}	HCO_2H, $NaHCO_3$	53.5	14	12
Sulfobus tokodaii	7.3	Mg^{2+}	$NaHCO_3$, Saturated CO_2	3.2	0.16	13
Sulfobus tokodaii	7.8	Mg^{2+}	Gaseous CO_2	46	2.3	14

[a] not specified

Reference

(1) Are, K. R. A.; Ohshima, S.; Koike, Y.; Asanuma, Y.; Kashikura, S.; Tamura, M.; Matsuda, T. Enzymatic Direct Carboxylation under Supercritical CO_2. *Biochem Eng J* **2021**, *171*, 108004. https://doi.org/10.1016/j.bej.2021.108004.

(2) Harding, C. J.; Cadby, I. T.; Moynihan, P. J.; Lovering, A. L. A Rotary Mechanism for Allostery in Bacterial Hybrid Malic Enzymes. *Nat Commun* **2021**, *12*, 1228. https://doi.org/10.1038/s41467-021-21528-2.

(3) Mandler, D.; Willner, L. Photochemical Fixation of Carbon Dioxide: Enzymic Photosynthesis of Malic, Aspartic, Isocitric, and Formic Acids in Artificial Media. *J Chem Soc Perkin Trans 2* **1988**, *7*, 997-1003. https://doi.org/10.1039/P29880000997.

(4) Inoue, H.; Yamachika, M.; Yoneyama, H. Photocatalytic Conversion of Lactic Acid to Malic Acid through Pyruvic Acid in the Presence of Malic Enzyme and Semiconductor Photocatalysts, *J Chem Soc* **1992**, *88*, 2215-2219. https://doi.org/10.1039/FT9928802215.

(5) Sequoia, E.; Sugimura, K.; Kuwabata, S.; Yoneyama, H. Electrochemical fixation of carbon dioxide in pyruvic acid to yield malic acid using malic enzyme as an electrocatalyst. *Bioelectrochem Bioenerg* **1990**, *24*, 241-24. https://doi.org/10.1016/0022-0728(90)87523-M.

(6) Itoh, T.; Asada, H.; Tobioka, K.; Kodera, Y.; Matsushima, A.; Hiroto, M.; Nishimura, H.; Kamachi, T.; Okura, I.; Inada, Y. Hydrogen Gas Evolution and Carbon Dioxide Fixation with Visible Light by Chlorophyllin Coupled with Polyethylene Glycol. *Bioconjug Chem* **2000**, *11*, 8–13. https://doi.org/10.1021/bc990045t.

(7) Ohno, Y.; Nakamori, T.; Zheng, H.; Suye, S. I. Reverse Reaction of Malic Enzyme for HCO_3^- Fixation into Pyruvic Acid to Synthesize L-Malic Acid with Enzymatic Coenzyme Regeneration. *Biosci Biotechnol Biochem* **2008**, *72*, 1278–1282. https://doi.org/10.1271/bbb.70772.

(8) Amao, Y.; Ishikawa, M. Visible Light and Enzymatic Induced Synthesis of Malic Acid from Pyruvic Acid and HCO_3^- with the Combination System of Zinc Chlorophyll Derivative and Malic Enzyme in Water Media. *Catal Commun* **2007**, *8*, 523–526. https://doi.org/10.1016/j.catcom.2006.07.026.

(9) Zheng, H.; Ohno, Y.; Nakamori, T.; Suye, S. ichiro. Production of L-Malic Acid with Fixation of HCO_3^- by Malic Enzyme-Catalyzed Reaction Based on Regeneration of Coenzyme on Electrode Modified by Layer-by-Layer Self-Assembly Method. *J Biosci Bioeng* **2009**, *107*, 16–20. https://doi.org/10.1016/j.jbiosc.2008.09.009.

(10) Ye, X.; Honda, K.; Morimoto, Y.; Okano, K.; Ohtake, H. Direct Conversion of Glucose to Malate by Synthetic Metabolic Engineering. *J Biotechnol* **2013**, *164*, 34–40. https://doi.org/10.1016/j.jbiotec.2012.11.011.

(11) Morimoto, Y.; Honda, K.; Ye, X.; Okano, K.; Ohtake, H. Directed Evolution of Thermotolerant

Malic Enzyme for Improved Malate Production. *J Biosci Bioeng* **2014**, *117*, 147–152. https://doi.org/10.1016/j.jbiosc.2013.07.005.

(12) Guo, X.; Liu, Y.; Wang, Q.; Wang, X.; Li, Q.; Liu, W.; Zhao, Z. K. Non-natural Cofactor and Formate-Driven Reductive Carboxylation of Pyruvate. *Angew Chem, Int Ed* **2020**, *132*, 3167–3170. https://doi.org/10.1002/ange.201915303.

(13) Takeuchi, M.; Amao, Y. Biocatalytic Fumarate Synthesis from Pyruvate and CO_2 as a Feedstock. *React Chem Eng* **2022**, *7*, 1931–1935. https://doi.org/10.1039/d2re00039c.

(14) Takeuchi, M.; Amao, Y. Visible-Light Driven Fumarate Synthesis from Pyruvate and Gaseous CO_2 with a Hybrid System of Photocatalytic NADH Regeneration and Dual Biocatalysts. *RSC Sus* **2023**, *1*, 1874–1882. https://doi.org/10.1039/d3su00194f.
